# Synthesis and Characterization of a Sustained Nitric Oxide-Releasing Orthodontic Elastomeric Chain for Antimicrobial Action

**DOI:** 10.3390/ijms25136982

**Published:** 2024-06-26

**Authors:** Alec McDonald, Carly Warden, Jinlian Tan, Kellianne M. Piell, Jill M. Steinbach-Rankins, Nandakumar Janakiraman, David A. Scott, Marsha P. Cole, Sudha Gudhimella

**Affiliations:** 1 Department of Orthodontics, University of Louisville School of Dentistry, Louisville, KY 40202, USA; alec.mcdonald@louisville.edu (A.M.); cewarden92@gmail.com (C.W.); 2 Department of Oral Immunology and Infectious Diseases, University of Louisville, Louisville, KY 40202, USA; jinlian.tan@louisville.edu (J.T.); david.scott@louisville.edu (D.A.S.); 3 Department of Biochemistry and Molecular Genetics, Louisville, KY 40202, USA; kellianne.piell@louisville.edu; 4 Department of Pharmacology and Toxicology, University of Louisville School of Medicine, Louisville, KY 40202, USA; jmstei01@louisville.edu; 5 Georgia School of Orthodontics, Atlanta, GA 30350, USA

**Keywords:** orthodontic brackets, S-Nitroso-N-Acetylpenicillamine (SNAP), cytotoxicity, orthodontic ligatures, dental plaque, antibacterial chains, elastomeric chainswhite spot lesions, nitric oxide, SNAP

## Abstract

The acidic byproducts of bacteria in plaque around orthodontic brackets contribute to white spot lesion (WSL) formation. Nitric oxide (NO) has antibacterial properties, hindering biofilm formation and inhibiting the growth of oral microbes. Materials that mimic NO release could prevent oral bacteria-related pathologies. This study aims to integrate S-nitroso-acetylpenicillamine (SNAP), a promising NO donor, into orthodontic elastomeric ligatures, apply an additional polymer coating, and evaluate the NO-release kinetics and antimicrobial activity against *Streptococus mutans*. SNAP was added to clear elastomeric chains (8 loops, 23 mm long) at three concentrations (50, 75, 100 mg/mL, and a control). Chains were then coated, via electrospinning, with additional polymer (Elastollan^®^) to aid in extending the NO release. NO flux was measured daily for 30 days. Samples with 75 mg/mL SNAP + Elastollan^®^ were tested against *S. mutans* for inhibition of biofilm formation on and around the chain. SNAP was successfully integrated into ligatures at each concentration. Only the 75 mg/mL SNAP chains maintained their elasticity. After polymer coating, samples exhibited a significant burst of NO on the first day, exceeding the machine’s reading capacity, which gradually decreased over 29 days. Ligatures also inhibited *S. mutans* growth and biofilm formation. Future research will assess their mechanical properties and cytotoxicity. This study presents a novel strategy to address white spot lesion (WSL) formation and bacterial-related pathologies by utilizing nitric oxide-releasing materials. Manufactured chains with antimicrobial properties provide a promising solution for orthodontic challenges, showing significant potential for academic-industrial collaboration and commercial viability.

## 1. Introduction

### 1.1. Oral Hygiene Barrier and Cariogenic Plaque

Obstructed oral hygiene is a consequence of orthodontic treatment with fixed appliances [1]. Therefore, treatment with fixed appliances may be a risk factor for plaque accumulation and associated pathologies [2,3,4]. Appliances, such as brackets, wires, coils, and ligatures, hinder access to tooth surfaces and make mechanical cleaning more complex. Additionally, the effectiveness of physiological self-cleansing by the tongue, cheeks, and saliva is reduced [5]. Moreover, after orthodontic treatment, a shift in the bacterial flora is often observed, resulting in higher concentrations of acidogenic bacteria, such as *S. mutans* and *Lactobacilli* [6,7]. The cariogenic potential of these microorganisms is strengthened by their ability to live and proliferate on hard, non-shedding tooth surfaces [8]. Increasing bacterial loads, coupled with sugar intake, cause plaque pH to drop as bacteria ferment dietary carbohydrates, such as sucrose, into acids, contributing to the reduction [8]. A reduced pH below the critical value (5.0–5.5) may lead to the demineralization of hydroxyapatite crystals in the tooth enamel, which eventually results in dental caries and white spot lesions (WSL) [9].

Enamel demineralization is one of the most common consequences of biofilm formation among patients receiving fixed orthodontic treatment, affecting 50% to 70% of patients [1,10,11]. According to a longitudinal clinical study conducted by Zachrisson et al., it was found that all 75 patients who underwent fixed orthodontic treatment exhibited higher mean values for visible plaque, inflammation, and gingival recession compared to their pre-treatment values [2]. These findings are consistent with a study by Boke et al., which also demonstrated significant increases in plaque buildup, gingival inflammation, and recession of gingiva after treatment [12]. Poor oral hygiene and increased plaque have been recognized as contributing factors to prolonged treatment time and compromised treatment outcomes, according to a retrospective study by Beckwith et al. [13]. The study collected data from 140 subjects who completed treatment with fixed appliances in five orthodontic offices. Progress notes with entries indicating less than “good” oral hygiene were linked to an additional two-thirds of a month in estimated treatment time [13]. Therefore, the introduction of antibacterial orthodontic appliances and accessories may help improve oral hygiene, reduce plaque accumulation, and shorten treatment duration.

### 1.2. White Spot Lesions

Enamel decalcification can become apparent around the fourth week of orthodontic treatment in cases of poor oral hygiene [14]. These decalcifications often manifest as white spot lesions (WSL) appearing as small chalky lines along the bracket periphery [15]. According to a meta-analysis by Venkatachalapathy et al., WSL occurrence in fixed orthodontic treatment is common, with an incidence rate of 45.8% and a prevalence rate of 68.4% [16]. White spot lesions (WSL) represent the first clinical evidence of demineralization and incipient lesions, which can potentially further progress into dentin as caries [17]. As such, it is crucial for orthodontists and dentists to address these lesions and their contributing factors before they become more destructive. Managing WSLs requires a multifactorial approach that includes preventing demineralization, reducing biofilm proliferation, and remineralizing lesions, as well as esthetic-focused approaches such as thinning, micro-abrasion, resin infiltration, and whitening [18].

Implementing effective oral hygiene is the foremost approach to addressing WSLs [19]. The use of adjunctive therapies to supplement insufficient plaque control has been studied, but their protocols and efficacy remain ambiguous [20,21]. Common to orthodontic practices, a one-time application of fluoride varnish prior to treatment did not provide any additional WSL prevention compared to good oral hygiene [19,20,21]. However, increasing the frequency of application has shown some success [22]. A Cochrane review found that applying fluoride varnish every six weeks during orthodontic treatment can aid in remineralization and prevent further demineralization [22]. In pursuit of sustained fluoride deposition, researchers have explored the use of fluoride-releasing bonding systems at the base of orthodontic brackets [23,24]. Several studies have shown that such systems effectively reduce enamel demineralization. Furthermore, it has been noted that fluoride-releasing adhesives do not compromise the bond strength of orthodontic brackets. However, further research is necessary to determine the long-term effects and cost-effectiveness of these adhesives.

Studies involving orthodontic elastomeric ligatures with fluoride-releasing properties have produced varying results. For instance, one study found that the use of fluoridated elastomers was ineffective in reducing streptococcal and/or anaerobic bacterial growth over a clinically relevant period [25]. This randomized, prospective, longitudinal clinical trial included 30 subjects and had a split-mouth crossover design. After a mean treatment period of 40 days, a logistic regression model was used to analyze the presence or absence of streptococcal or anaerobic bacteria in relation to the use of fluoridated elastomers. Results revealed that the use of fluoridated elastomers did not significantly reduce either streptococcal (*p* = 0.288) or anaerobic bacterial counts (*p* = 0.230) [25]. In a study by Miura et al., it was demonstrated that the use of fluoridated elastomers was insufficient at preventing enamel decalcification [26]. Additionally, it was suggested the incorporation of fluoride may negatively impact the elastomer’s physical properties, resulting in faster deterioration [27,28,29]. It is generally regarded that the anticaries action of fluoride is related to remineralization, rather than reducing the overall number and concentration of cariogenic bacteria [30,31]. Perhaps a shift in adjunctive therapies and appliances should be directed towards the etiology of the demineralization and the introduction of effective means to reduce biofilm populations, rather than targeting the palliative measures of fluoride at the enamel surfaces.

### 1.3. Biological Implications of Nitric Oxide

Until the late 1980s, nitric oxide (NO) was primarily viewed as an environmental pollutant, generated by industrial processes, automobile exhausts, and electrical storms, as well as a toxic component of cigarette smoke [32,33,34,35]. However, Ignarro and Furchgott’s research in the 1980s recognized NO’s role in physiology, revealing it to be the endothelium-derived relaxing factor responsible for vasodilation and blood pressure regulation [36,37,38]. Since then, researchers have uncovered numerous functions of nitric oxide and acknowledge NO’s role in preventing platelet activation and adhesion, inhibiting bacterial proliferation and biofilm formation, and facilitating signaling within the immune system [33,39,40,41]. Nitric oxide’s antibacterial properties make it a promising candidate for future medical device development. NO exhibits both nitrosative and oxidative effects, producing a range of reactive species that disrupt cellular functions and structures by interacting with microbial proteins, DNA, and enzymes [33,42]. At extreme concentrations, the cytotoxic effects of nitric oxide may become apparent, largely dependent on its reactivity with various radicals, more specifically superoxide (O_2•_^−^) in the formation of peroxynitrite (ONOO^−^) [43]. Peroxynitrite is a highly reactive nitrogen species (RNS) that can cause significant damage to cellular components, including lipids, proteins, and DNA [44]. It has been shown that peroxynitrite may cause oxidative stress and tissue damage, as well as dysregulation of cellular signaling and impaired mitochondrial function [45,46,47]. Yet, NO is generally considered safe at lower concentrations due to controlled dosing and targeted delivery [48]. Furthermore, nitric oxide has demonstrated effective activity against both Gram-positive and Gram-negative bacteria, such as *Staphylococcus aureus*, *Escherichia coli*, and *Pseudomonas aeruginosa* [49].

Therapies leveraging NO’s actions could prove beneficial for those with associated health complications and diseases, including high blood pressure, hypertension, endothelial dysfunction, and bacteria-related pathologies [50,51]. Biomedical researchers are interested in developing modified materials for the external delivery of nitric oxide (NO) to specific sites [52]. The nitric oxide donor, S-nitroso-acetylpenicillamine (SNAP), is acclaimed for its ability to integrate into elastomeric materials like silicones and polyurethanes while exhibiting long-term stability and retention following sterilization techniques [53,54,55] Nitric oxide is released from these materials through processes such as thermal decomposition, metal catalysis, or exposure to light [56]. Recent studies involving the incorporation of SNAP into catheters and various medical devices have produced encouraging results by displaying antimicrobial defense against *S*. *aureus*, *Staphylococcus epidermidis*, and *Proteus mirabilis* [57,58].

### 1.4. Previous Findings and Addition of Polymer

In the first part of this research, carried out by Warden et al., orthodontic elastomeric chains (6 loops, 17 mm long) were treated with 75 mg of SNAP using the swell–encapsulation–shrink method [59]. Initially, three solutions with concentrations of 75, 125, and 200 mg/mL were developed, along with controls. However, once washed and dried, only the ligatures soaked in the 75 mg/mL SNAP solution, as well as the controls, maintained their elastic properties. Therefore, the study continued using only the 75 mg/mL SNAP chains. Following successful impregnation of the SNAP, NO flux was measured daily for 2 h/day for 4 days to determine the duration and magnitude of NO release. The samples demonstrated nitric oxide release for three days and showed no release over baseline on the fourth day [59]. The experimental chains in Warden’s study also demonstrated a good level of efficacy in defending against the oral pathogen *S. mutans* [59]. Biofilm formation on surfaces adjacent to the ligatures and planktonic growth in solution were both significantly decreased with experimental chains compared to controls (*p* < 0.05) [59]. While the chains demonstrated bactericidal effects and nitric oxide release, it is important to note that most common orthodontic practices replace patients’ elastomeric ligatures every 4–6 weeks [60]. Therefore, for this application to become more relevant in clinical settings, the NO-release period should be further extended. Orthodontic elastomeric chains demonstrating NO release for several weeks to a month may be more appropriate. Sustained nitric oxide release can be achieved in two ways: (1) by interfering with the factors initiating the NO release from SNAP and (2) by introducing a barrier to prevent NO from escaping. Since SNAP’s nitric oxide-donating capabilities are driven by light, it can be inferred that limiting the amount of light contacting the SNAP-impregnated chain may result in less expulsion of NO [56].

Application of an additional polymer coating to the SNAP-impregnated ligatures may provide a means for sustained release of nitric oxide. Elastollan^®^ (BASF, Ludwigshafen, Germany) is a thermoplastic polyurethane used in various commercial products due to its favorable properties, such as high wear and abrasion resistance, high tensile strength, and resistance to tear propagation, making it a viable option in orthodontic materials [61]. Coating the SNAP-impregnated ligatures with fine polymer fibers through controlled dispensing techniques like electrospinning could enable predictable and consistent amounts of coating. Electrospinning, a voltage-driven fabrication process, utilizes a specific electrohydrodynamic phenomenon to produce small fibers from a polymer solution [62]. The technology has been successfully used to generate fibers from various materials, including natural and synthetic polymers in the form of solution or melt [63].

With the deficient amount of literature regarding the integration of nitric oxide-releasing compounds for antibacterial action in orthodontics, we aimed to expand upon the work of Warden et al. in a combined attempt to fabricate an antibacterial elastomeric chain of clinical relevance. In this study, we hypothesize that incorporating the NO-donor molecule, S-nitroso-acetylpenicillamine, into existing orthodontic elastomeric ligatures and applying an additional polymer coating via electrospinning will result in a sustained release of nitric oxide (NO) over a thirty-day period. Furthermore, we predict that these modified ligatures will exhibit antimicrobial defense effects against *Streptococcus mutans*.

## 2. Results

### 2.1. SNAP Impregnation

**Figure 1:**
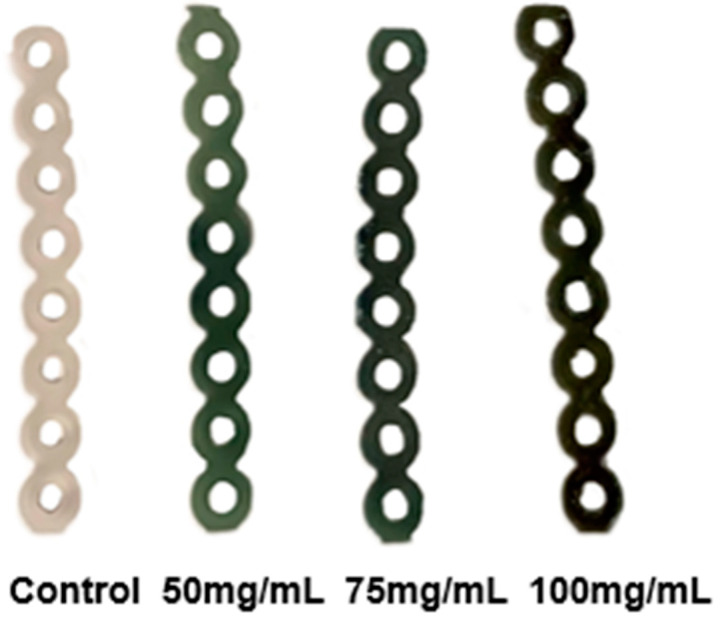
Experimental chains following SNAP impregnation demonstrating a light-to-dark gradient of green color.

**Figure 2:**
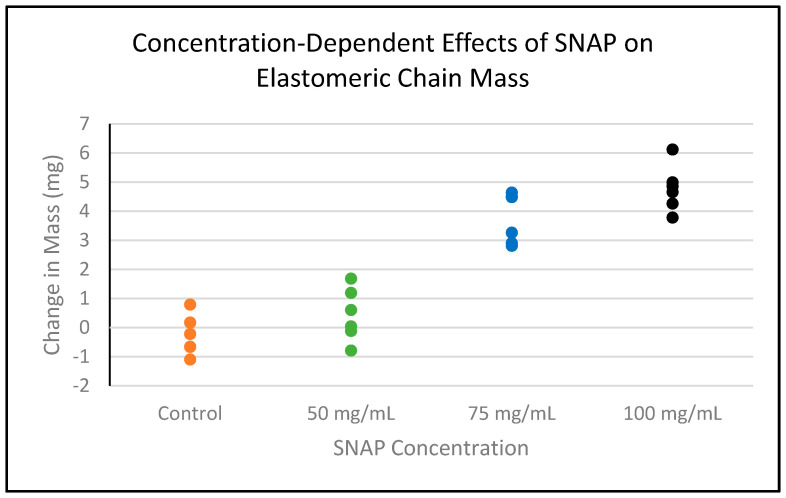
Scatterplot showing the relationship between different concentrations of SNAP and the corresponding changes in chain mass after drying. The Spearman correlation coefficient was found to be 0.88 (*p* < 0.001), indicating a strong positive correlation.

SNAP was successfully incorporated into existing clear elastomeric ligatures at three concentrations. All experimental and control samples returned to the same dimensions after drying and experimental chains exhibited a light-to-dark gradient of green color from lowest to highest concentration, respectively (Figure 1). The masses of ligatures were measured prior to and following impregnation (Table 1). The Spearman correlation coefficient was calculated to evaluate the strength of the relationship between the concentration of added SNAP and the change in chain mass. The coefficient was found to be 0.88 (*p* < 0.001), indicating a strong positive correlation between the two variables (Figure 2). The controls exhibited no increase in mass. In part 1 of this study, conducted by Warden et al., chains soaked in concentrations of 125 and 200 mg/mL SNAP/THF broke under tensile stress [59]. Ligatures incubated in 75 mg/mL solution as well as control chains maintained their elastic properties; therefore, in this experiment, all remaining procedures were carried out using the 75 mg/mL SNAP/THF samples.

### 2.2. Polymer Electrospinning

**Figure 3:**
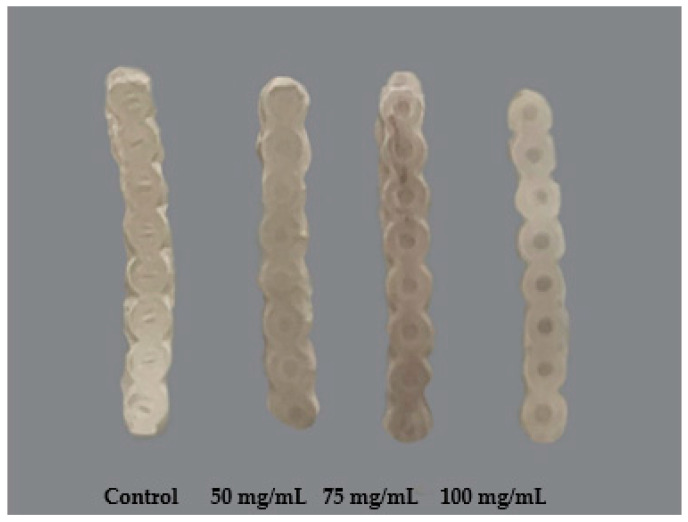
Elastollan^®^ coated experimental chains.

Experimental chains (75 mg/mL SNAP/THF) were successfully coated with the Elastollan^®^ polymer solution (Figure 3). Once dried, the samples exhibited similar stretch compared to their non-coated predecessors and controls.

### 2.3. Nitric Oxide Release Testing

**Figure 4:**
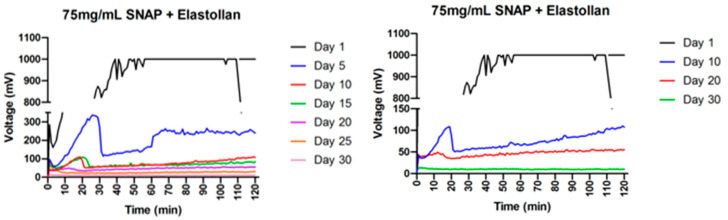
NO-release measurements. Every 5-day reading (**left**), every 10-day reading (**right**).

The experimental ligature (75 mg/mL SNAP/THF + Elastollan^®^) exhibited a large burst of NO release followed by a sharp decrease and then steady-state NO flux at 2 h on each day of testing with the NOA (Figure 4). On the first day of testing, the chain exhibited NO released above the reading capability of the machine and maintained this reading. This flux gradually decreased for twenty-nine days until showing no NO release over baseline on the thirtieth day of testing.

### 2.4. Bacterial Testing

#### 2.4.1. Planktonic Growth in Surrounding Solution

**Figure 5:**
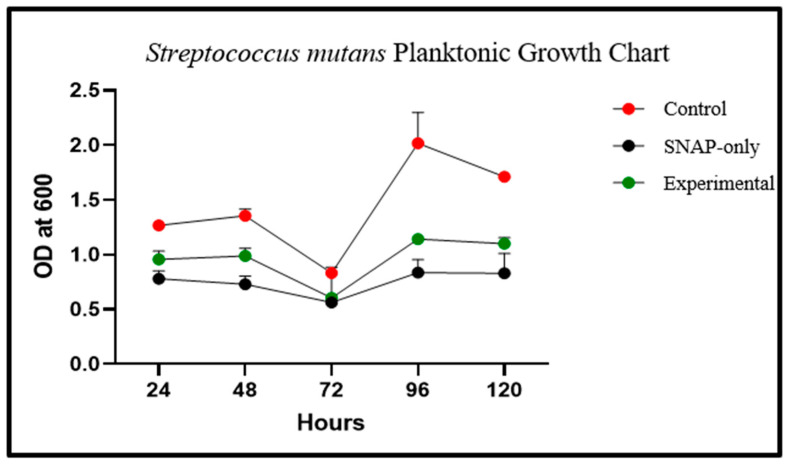
*S. mutans* planktonic growth curves measured at 24, 48, 72, 96, and 120 h. SNAP-only chains were impregnated with 75 mg/mL SNAP/THF. Experimental chains were impregnated with 75 mg/mL SNAP/THF and coated with Elastollan^®^.

Growth curves in solutions containing experimental samples or SNAP-only ligatures vs. control exhibited markedly lower growth by 24 h and inhibition was maintained for 120 h (Figure 5). On average, at 24 h, vials containing experimental chains experienced a 24.39% decrease in planktonic growth compared to control chains. At 120 h, vials containing experimental ligatures experienced a 35.56% decrease compared to controls.

#### 2.4.2. Biofilm Formation on Surrounding Surfaces

**Figure 6:**
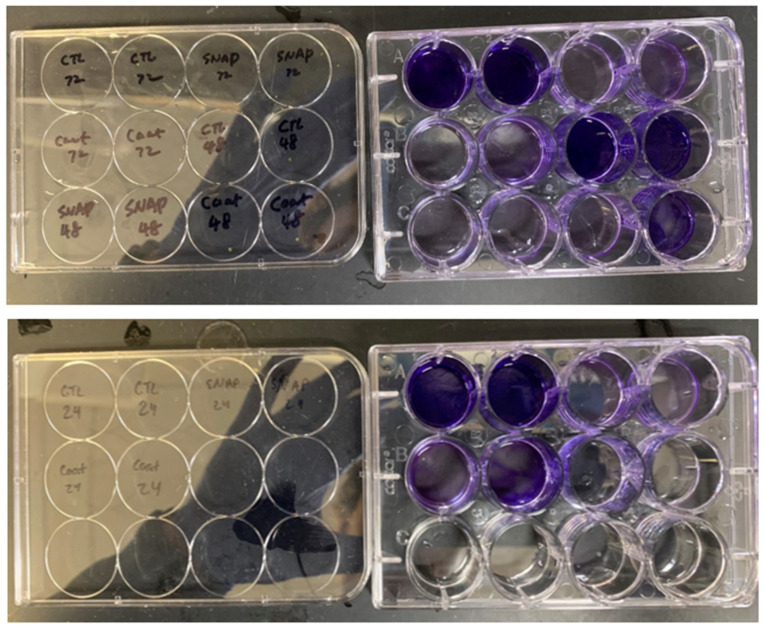
Twelve-well plate with *S. mutans* biofilms and crystal violet staining. All tests were performed in duplicate.

**Figure 7:**
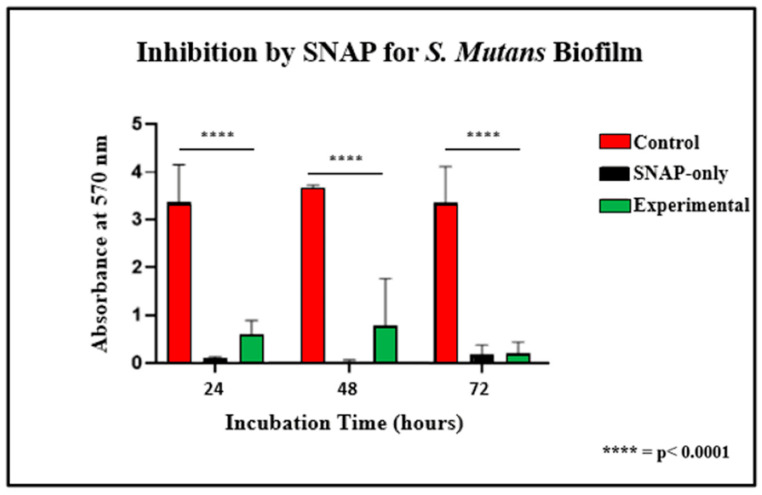
Biofilm formation on surfaces adjacent to the chains at 24, 48, and 72 h.

After plating 12-well plates with *S. mutans* biofilm and staining with crystal violet, wells containing experimental samples or SNAP-only chains exhibited a notable reduction in biofilm formation compared to controls (Figure 6). Statistical analysis revealed a significant decrease in biofilm formation on the well surface for both experimental and SNAP-only chains at 24, 48, and 72 h (*p* < 0.0001) (Figure 7). Additionally, there was no statistically significant difference in biofilm formation on the wells’ surfaces between those containing a coated experimental chain and those containing a SNAP-only chain. This indicates that the additional coating did not interfere with the bactericidal activity.

## 3. Discussion

Maintaining proper oral hygiene practices with fixed orthodontic appliances can be challenging and, if overlooked, may lead to various detrimental effects on the teeth and supporting structures. The rough and irregular surfaces of the fixed appliances create new areas for bacterial adhesion and proliferation, making brushing and flossing more complex [5,64]. These bacteria can multiply and release harmful metabolic byproducts that can damage the enamel surface and make the teeth more susceptible to irreversible harm. The initial sign of such damage appears as white spot lesions (WSLs), which have a white chalky appearance and are caused by enamel decalcification resulting from the acidic metabolites of fermentable sugars by bacteria. While esthetically unfavorable, WSLs also provide a window for further irreversible tooth damage. If left untreated, the incipient lesion may progress to the underlying dentin and result in dental caries and cavitation. Addressing WSLs promptly can help prevent more severe dental problems from developing [17].

Efforts to increase oral hygiene literacy and adjunctive oral hygiene practices in patients with fixed orthodontic appliances have been implemented but their effectiveness remains uncertain [24,25]. Fluoridated bonding agents and elastomers can serve as corrective measures aimed at remineralizing damaged enamel; however, targeting the etiology of white spot lesions rather than remedial therapies may be more beneficial. As a key contributor responsible for the development of white spot lesions, *Streptococcus mutans* demonstrates susceptibility to the endogenously produced compound, nitric oxide (NO) [33,39]. Nitric oxide displays a variety of physiological functions, including roles in vasodilation and bacterial suppression [39]. Research exploring the biomedical use of nitric oxide’s antibacterial properties has been conducted but is deficient in dentistry and orthodontics [54,56,57,58]. Introducing materials with antibacterial properties of NO offers the potential to suppress bacterial-related pathologies, such as white spot lesions.

S-nitroso-acetylpenicillamine (SNAP) is a nitric oxide donor, responsive to light, that can be incorporated into rubber-like materials, including silicones and polyurethanes, and has effectively demonstrated bacterial suppression in studies [43,54,56,57,65,66]. By incorporating SNAP into orthodontic elastomeric ligatures and subjecting them to ordinary light exposure, significant nitric oxide release can occur, effectively repressing bacterial proliferation. However, the efficacy of these chains depends on several factors, such as the amount of SNAP loaded into the material, SNAP’s degradation rate, and the susceptibility of the microbe to the nitric oxide molecule. In this study, experimental orthodontic ligatures were impregnated with 75 mg of SNAP/THF, coated with an additional Elastollan^®^ polymer, and tested for their nitric oxide-releasing capabilities and antibacterial effectiveness. This study builds upon Part 1 of the initial research conducted by Warden et al., which demonstrated the release of nitric oxide from elastomeric ligatures for three consecutive days [59]. The experimental samples in this study were fabricated with the intent of sustained nitric oxide release. Compared to the control chains, the coated experimental samples in this study demonstrated nitric oxide release for twenty-nine days until showing no release over baseline on the thirtieth day. The experimental ligatures utilized in this study exhibited a significant reduction in *Streptococcus mutans* biofilm production on surfaces adjacent to the chains compared to their controls (*p* < 0.0001). Additionally, solutions containing the experimental samples exhibited reduced *S. mutans* planktonic growth. Moreover, these ligatures exhibited no statistically significant differences in biofilm inhibition compared to their non-coated predecessors from Part 1 completed by Warden et al. [59]. These data suggest that the addition of a polymer coating did not limit the antibacterial effectiveness to a sub-functional status. The rise in optical density (OD600) readings at 96 h may be indicative of the regrowth of residual bacteria. Future studies should investigate the effect of subculturing *S. mutans* as time progresses, followed by OD600 measurement.

If the strength and elasticity of these experimental ligatures remain uncompromised, this technology could have the potential to reduce the prevalence of plaque-mediated pathologies associated with fixed orthodontic appliances. By incorporating compounds that release products naturally found in human physiology, such as nitric oxide, greater biocompatibility and patient acceptance may be achieved. A limitation of this study included only characterizing the functions of chains impregnated with one concentration of SNAP. Given more time to conduct this study, samples of varying concentrations should be prepared and tested for their nitric oxide expulsion and antibacterial effectiveness, as well. The experimental sample in this study was chosen to remain consistent with the findings of Warden et al. [59]. In their study, it was noted that elastomeric chains soaked in higher concentrations of 125 and 200 mg/mL SNAP broke under tensile stress; therefore, the remainder of their data was gathered using ligatures composed of 75 mg/mL of SNAP. Also, adjustments to the thickness of the Elastollan^®^ polymer coating could yield different NO and antibacterial effects. However, it is important to maintain a thickness that still renders the elastomeric chain functionable in orthodontics.

Finally, the future goals of this project are aimed at assessing the functional capacity of the ligatures in an orthodontic setting as well as evaluating their biocompatibility in the oral cavity. Future goals also include testing their antibacterial action against other common oral microbes, such as *Porphyromonas gingivalis* and *Streptococcus gordonii*, as well as testing antibacterial action for a longer period, such as thirty days. An antibacterial assessment of longer duration may reinforce the nitric oxide expulsion data revealed in this study. As the experimental chains in this study released quantitative amounts of nitric oxide for twenty-nine days, it is our aim for bacterial growth to be stunted for a period that parallels this duration.

## 4. Materials and Methods

The following methods for SNAP Impregnation, Nitric Oxide Release Testing, and Bacterial Testing were adapted from Warden et al. [59].

### 4.1. SNAP Impregnation

**Figure 8:**
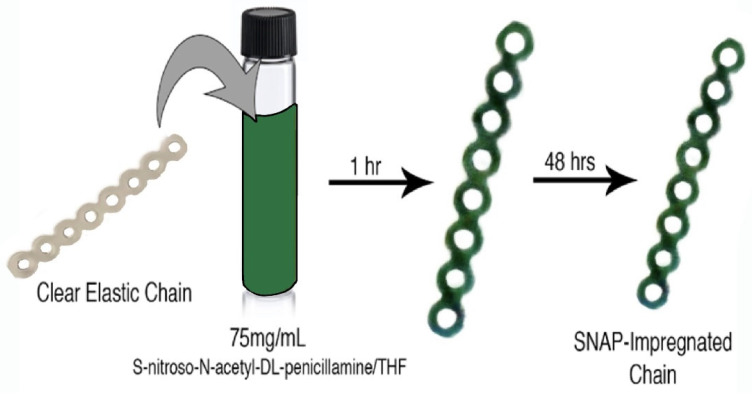
Schematic of swell–encapsulation–shrink method for fabricating chains.

**Figure 9:**
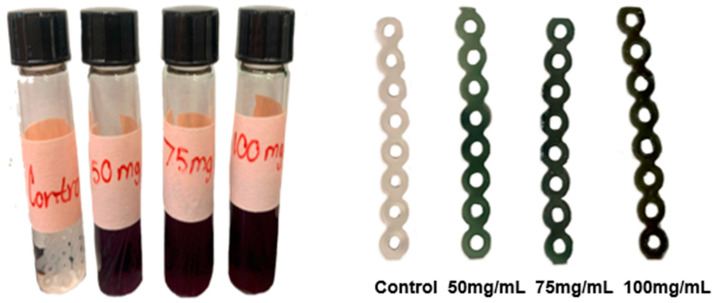
Chains incubating in SNAP + THF solutions at varying concentrations (**left**). SNAP-impregnated chains at varying solutions (**right**).

S-nitroso-acetylpenicillamine (Pharmablock, Shanghai, China) was incorporated into an existing clear elastomeric ligature (8 loops, 23 mm long; J-120 Closed Clear Energy Chain, Rocky Mountain Orthodontics, Denver, CO, USA) using a swell–encapsulation–shrink method (Figure 8). SNAP was dissolved in 5 mL glass vials containing 4 mL tetrahydrofuran (THF) (Sigma-Aldrich, St. Louis, MO, USA) to produce three solutions at concentrations of 50, 75, and 100 mg/mL, respectively. Control vials contained 4 mL THF without SNAP. Both experimental and control ligatures were immersed in these solutions for 1 h, then removed, washed with PBS buffer (Fisher Scientific, Waltham, MA, USA), and left to dry in darkness for 48 h within a fume hood. Successful impregnation was visually confirmed by observing the characteristic green color of SNAP (Figure 9). The masses of the samples were measured before and after impregnation. The Spearman correlation coefficient was then calculated to assess the strength of the relationship between the concentration of added SNAP and the change in chain mass. Subsequently, elastomeric chains were stored in dark, dry aluminum foil wrappings.

### 4.2. Polymer Electrospinning

**Figure 10:**
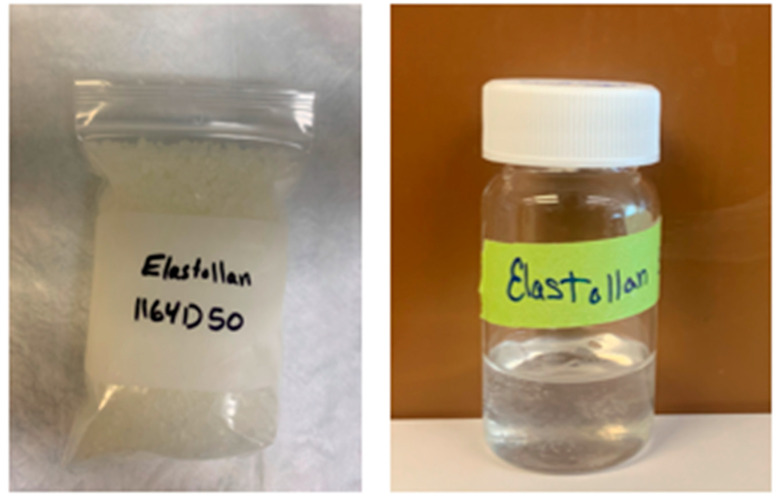
Elastollan^®^ polymer pellets (**left**) and final polymer solution (**right**).

Via electrospinning, experimental ligatures (75 mg/mL SNAP/THF) were coated with an additional Elastollan^®^ (BASF, Ludwigshafen, Germany) polymer matrix. This concentration was chosen to remain consistent with Warden et al.’s study [59]. Given the opportunity, samples of 50 mg/mL and 100 mg/mL were coated and tested, as well. A polymer solution of Elastollan^®^ was produced by weighing out 10% *w*/*v*% polymer (100 mg Elastollan^®^ in 1 mL solvent) dissolved in THF:DMF 1:1 *v*:*v* (Figure 10; Sigma-Aldrich, St. Louis, MO, USA). The solution was incubated at 37 °C overnight.

**Figure 11:**
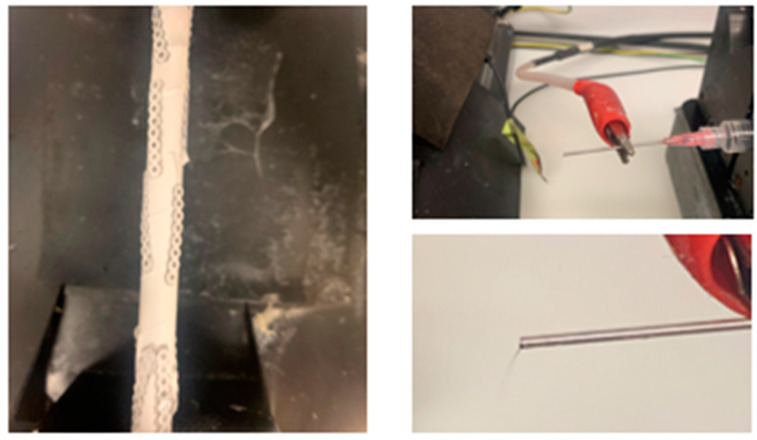
Photographs of the chains on the rotating rod (**left**) and polymer threads dispensing from the syringe (**right**).

**Figure 12:**
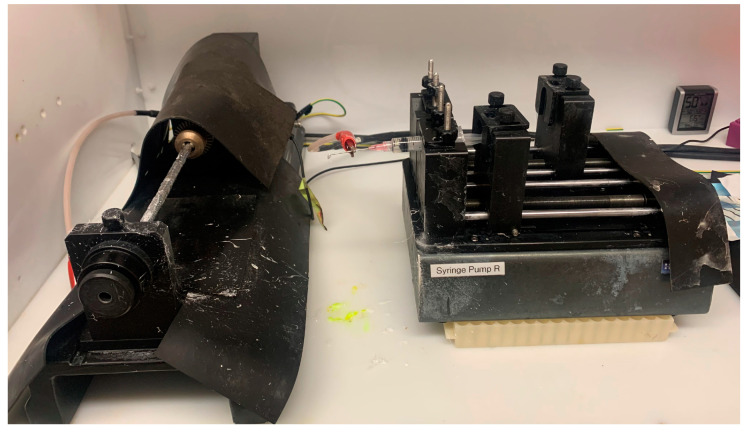
Electrospinning polymer coating to SNAP-impregnated chains. Pictured is the rotating rod housing the chains (**left**) and the syringe dispenser containing the polymer solution (**right**).

On the day of spinning, 6 mL of polymer solution was transferred to a plastic syringe with needle attached (Becton Dickinson, Franklin Lakes, NJ, USA). The syringe was emptied of all air bubbles and attached firmly to an automatic dispenser (Figure 11). Experimental ligatures were adhered to a mandrel located 15 cm from the syringe needle tip (Figure 12). The flow rate of the dispenser was set to 6 mL/h. and mandrel set to 300 rpm. The electrospinning was completed under an electric field of 20 kV. After 30 min, the electrospinning process was paused, allowing the chains to dry until flipped. They then ran for an additional 30 min coating the other side. The ligatures were dried and removed. All electrospinning was completed in the dark.

### 4.3. Nitric Oxide Release Testing

**Figure 13:**
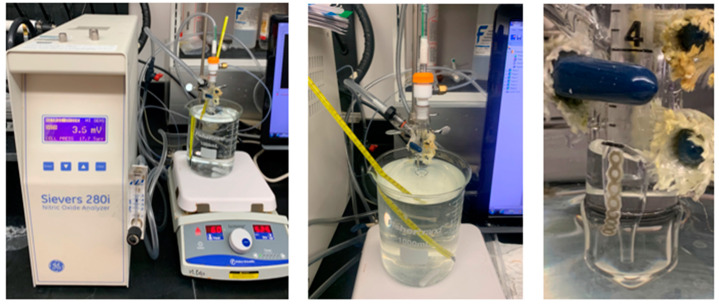
Sievers Chemiluminescence Nitric Oxide Analyzer (**left**), NO-release measurement configuration (**middle**), and chains suspended in PBS (**right)**.

Nitric Oxide flux from a single ligature (from the 75 mg/mL SNAP/THF + Elastollan^®^) was measured daily for 2 h/day for 30 days using a Sievers Chemiluminescence Nitric Oxide Analyzer (GE Analytical Instruments, Boulder, CO, USA) to determine duration and magnitude of NO release (Figure 13). The chain sample was submerged in a glass container with 4 mL of PBS within a water bath set at 37 °C. Nitric oxide released from the sample was then transported into the detection chamber (Figure 13). Readings were conducted until a consistent flux was attained. During periods of non-testing, ligatures were kept in amber glass vials with 1 mL of PBS at 37 °C.

### 4.4. Bacterial Testing

The bactericidal properties of the obtained sample (75 mg/mL SNAP/THF + Elastollan^®^) were tested against the common oral microbe, *S. mutans*, in two separate microbial studies to determine planktonic growth surrounding the chain and biofilm formation on the ligature itself. Control samples and ligatures impregnated with 75 mg of SNAP only were tested, as well [59].

### 4.5. Planktonic Growth in Surrounding Solution

**Figure 14:**
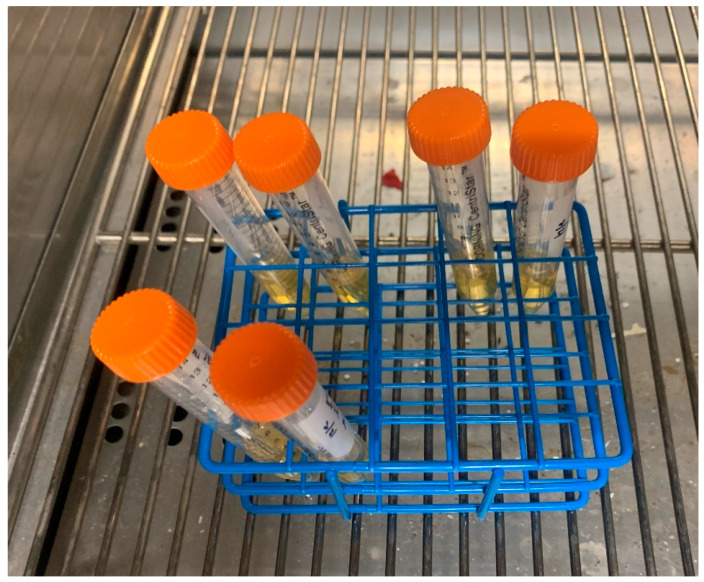
*S. mutans* in 4 mL solution of BHI + 2% sucrose. (**Top left vials**) control chains, (**top right vials**) chains impregnated with 75 mg/mL SNAP/THF, (**bottom left**) experimental chains (75 mg/mL SNAP/THF + Elastollan^®^).

A standardized solution of KPSP2 *Streptococcus mutans* (American Type Culture Collection, Manasses, VA, USA) in brain–heart infusion (BHI; Becton Dickinson, Franklin Lakes, NJ, USA) was added to a vial with 4 mL solution of BHI + 2% sucrose (Figure 14). The ratio of inoculant to media was 1:30. Each vial contained either (1) two control chains, (2) two chains impregnated with 75 mg/mL SNAP/THF, or (3) two coated experimental chains (75 mg/mL SNAP/THF + Elastollan^®^). All tests were performed in duplicate totaling 6 vials. Vials were incubated at 37 °C, aerobically, without shaking, in the dark. Then, 1 mL of solution was removed at 24, 48, 72, 96, and 120 h, and optical density at 600 nm was measured using Eppendorf BioPhotometer Plus (Eppendorf AG, Hamburg, Germany) and recorded.

### 4.6. Biofilm Formation on Surrounding Surfaces

**Figure 15:**
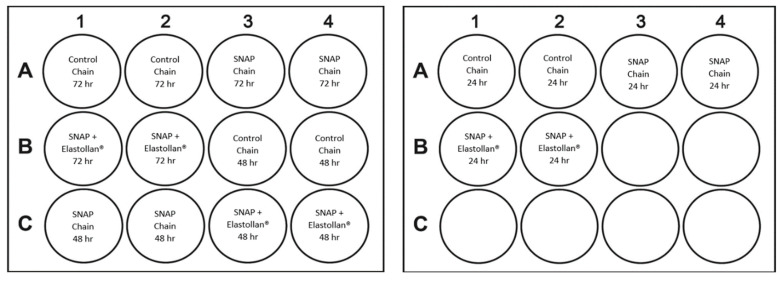
Biofilm on surrounding surfaces 12-well plate schematic.

In a 12-well plate, a standardized solution of *S. mutans* was added to a 1 mL solution of BHI supplemented with 2% sucrose. The ratio of Inoculant to media was 1:30. Experimental, SNAP-only, and control chains (1 chain/well) were suspended in the solution and incubated at 37 °C, aerobically, for periods of 24, 48, and 72 h (Figure 15). All samples were removed, wells were washed with 2 mL PBS, and 500 µL 0.1% crystal violet stain (0.3% stock; Becton Dickinson, Franklin Lakes, NJ, USA) was added to each well. Following incubation for 30 min, wells were washed again with water and allowed to dry. Then, 1 mL 30% acetic acid (MilliporeSigma, Burlington, MA, USA) was added to each well to dissolve the crystal violet, and absorbance was measured at 570 nm. All tests were performed in duplicate.

## 5. Conclusions

The nitric oxide-releasing molecule, S-nitroso-acetylpenicillamine, can be successfully incorporated into an existing orthodontic elastomeric ligature. Following impregnation, samples exhibited NO release over a twenty-nine-day period after the addition of a polymer coating. Ligatures demonstrating successful NO release inhibited both *S.coccus mutans* planktonic growth and growth on surfaces adjacent to the chain. Manufactured chains integrated with antimicrobial properties present a promising solution for combating white spot lesions and other bacterial-related issues in orthodontics. Furthermore, the advancement of these chains creates opportunities for collaboration between academia and industry, as well as the significant potential for commercial viability.

## Figures and Tables

**Table 1:** Average mass of an elastomeric chain (n = 6) before and after the addition of SNAP. Control chains were incubated in THF and dried under hood for 48 h.

	Before (mg)	After (mg)	% Change
Control (n = 6)	22.65 (SD = 0.63)	22.17 (SD = 0.55)	−2.12
50 mg/mL (n = 6)	22.50 (SD = 0.71)	24.94 (SD = 0.53)	+10.84
75 mg/mL (n = 6)	22.72 (SD = 0.77)	26.38 (SD = 0.77)	+16.11
100 mg/mL (n = 6)	22.42 (SD = 0.75)	27.06 (SD = 0.70)	+20.70

## Data Availability

The data that support the findings of this study are available from the corresponding author upon reasonable request.
